# Challenging Differential Diagnosis of Paroxysmal Atrial Fibrillation Versus Monomorphic Ventricular Tachycardia in an Elderly Woman: Application of Vereckei and Brugada Criteria

**DOI:** 10.7759/cureus.97568

**Published:** 2025-11-23

**Authors:** Jesse O'Rorke, Greyson Butler, Henry Zimmerman

**Affiliations:** 1 Osteopathic Medicine, Lake Erie College of Osteopathic Medicine, Bradenton, USA; 2 Emergency Medicine, Lee Health, Fort Myers, USA

**Keywords:** brugada criteria, synchronized cardioversion, ventricular tachycardia (vt), vereckei criteria, wide complex atrial fibrillation

## Abstract

Ventricular tachycardia (VT) is a potentially fatal arrhythmia that must be promptly distinguished from supraventricular tachycardia with aberrancy, including wide-complex atrial fibrillation with rapid ventricular response. Diagnostic algorithms such as the Brugada and Vereckei criteria aid clinicians in differentiating these rhythms and guiding urgent management. Chronic myocardial ischemia is a well-recognized substrate for VT due to scar formation and reentrant conduction pathways. We report the case of an 85-year-old woman with a history of coronary artery disease (CAD) and prior stenting who presented with dizziness, palpitations, hypotension, and wide-complex tachycardia. The initial differential diagnosis included VT vs. wide-complex atrial fibrillation. Application of the Brugada and Vereckei criteria supported the diagnosis of VT. The patient underwent successful synchronized cardioversion and was started on intravenous amiodarone, later transitioned to oral therapy. Further evaluation revealed complete thrombotic occlusion of the left circumflex artery consistent with chronic ischemic disease. Echocardiography demonstrated reduced ejection fraction and structural changes indicative of prior infarction. After cardioversion, she remained in sinus rhythm but exhibited QT interval prolongation. Troponin elevation was deemed nondiagnostic in the setting of recent cardioversion. She was discharged on optimal medical therapy for CAD with outpatient follow-up for possible implantable cardioverter-defibrillator evaluation. This case underscores the importance of applying validated criteria to distinguish VT from other wide-complex tachycardias, recognizing chronic ischemia as an arrhythmogenic substrate, and considering pharmacologic nuances of antiarrhythmic therapy. Amiodarone remains a cornerstone in VT management due to its broad efficacy and relative hepatic safety, but requires careful monitoring for QT interval prolongation and avoidance of interacting agents. Prompt rhythm identification, recognition of ischemic mechanisms, and individualized therapy are essential to improving outcomes in elderly patients with structural heart disease presenting with sustained VT.

## Introduction

Ventricular tachycardia (VT) is a potentially fatal arrhythmia that requires rapid identification and treatment [1]. Differentiating VT from supraventricular tachycardia (SVT) with aberrancy, including wide-complex atrial fibrillation with rapid ventricular response secondary to bundle branch block or preexcitation, is essential in guiding acute management. Clinical algorithms such as the Brugada and Vereckei criteria provide structured approaches to this distinction in the emergency setting [2,3]. Chronic myocardial ischemia is a well-established substrate for VT, promoting scar formation and reentrant conduction pathways even years after an initial coronary event. The incidence of VT increases with the degree of left ventricular dysfunction and myocardial fibrosis, both of which contribute to electrical instability and reentrant arrhythmogenesis. Pharmacologic management frequently includes amiodarone, which is unique among antiarrhythmics for lacking hepatotoxicity but carries the important risk of QT interval prolongation. We present a case of sustained monomorphic VT in an elderly patient with chronic ischemic occlusion, highlighting the role of diagnostic criteria, ischemic substrate, and the therapeutic considerations of amiodarone use.

## Case presentation

An 85-year-old woman with a past medical history of paroxysmal atrial fibrillation, transient ischemic attack, coronary artery disease (CAD), hypertension, and hyperlipidemia presented to the emergency department with a chief complaint of dizziness and palpitations. She stated that at 8:00 PM, the previous evening, she became severely dizzy, describing the feeling as the room spinning around her. She spent the night on the couch as she was too dizzy to stand and walk to her bedroom. She denied chest pain, overt syncope, or known fevers associated with the event. Emergency medical services reported that they found the patient to be confused, experiencing hypotension, a fever reaching 39°C, and wide complex tachycardia on her electrocardiogram (EKG).

**Figure 1:**
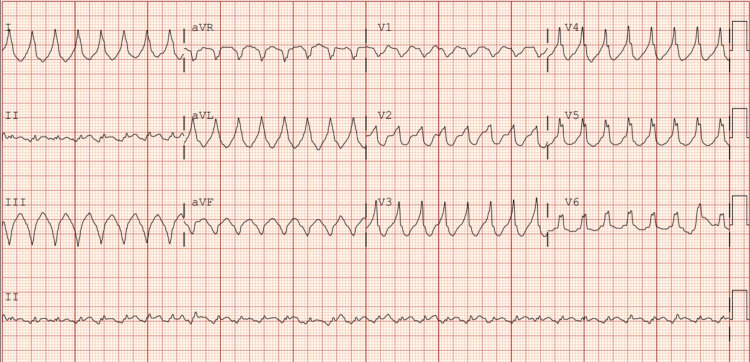
Electrocardiogram on presentation to the emergency department, demonstrating ventricular tachycardia aVR: augmented vector right; aVL: augmented vector left; aVF: augmented vector foot

Her initial blood pressure in the emergency department was 91/55 mmHg with a heart rate of 170 beats per minute. The EKG obtained demonstrated a wide-complex tachycardia at a rate of 190 beats per minute with no diagnostic changes of acute ischemia, as shown in Figure 1. Initial discussion between emergency department staff was whether the patient was in a rhythm indicative of monomorphic VT or wide complex rapid atrial fibrillation. Application of the Vereckei criteria revealed an initial Q wave in lead augmented vector right (aVR) measuring greater than 40 milliseconds, while the Brugada criteria demonstrated an R-S complex in lead V2 exceeding 100 ms in duration. Both findings support the conclusion, based on the Vereckei and Brugada algorithms, that the rhythm was consistent with VT.

**Figure 2:**
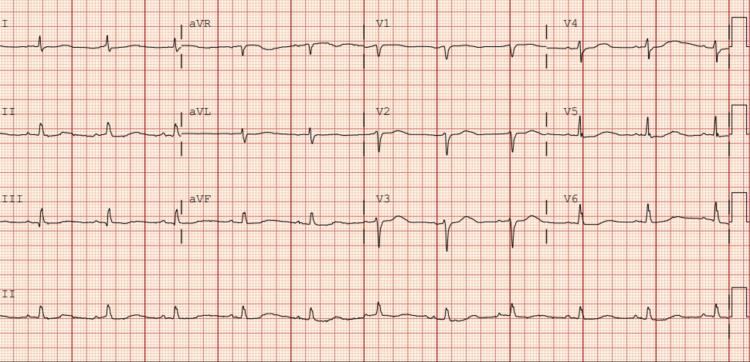
Electrocardiogram after cardioversion in the emergency department, demonstrating normal sinus rhythm with a prolonged QT interval aVR: augmented vector right; aVL: augmented vector left; aVF: augmented vector foot

After discussion with the patient and her family at bedside, she was emergently cardioverted using ketamine for sedation with good results and no complications. She remained in sinus rhythm from this point forward and was given an amiodarone infusion and bolus. Given the patient’s hypotension, ketamine was chosen as the sedative agent because it maintains cardiovascular stability relative to other sedatives that may exacerbate hypotension. On multiple reevaluations in the emergency department, she was hemodynamically stable and asymptomatic. Repeat EKG demonstrated normal sinus rhythm at a rate of 65 beats per minute with a prolonged QT interval without changes diagnostic for acute ischemia, as shown in Figure 2.

Blood cultures were obtained in the emergency department, and the patient was medicated with piperacillin-tazobactam. She was hydrated with a liter of normal saline and then given an additional 500 mL of normal saline due to her presenting acute kidney injury, with a creatinine of 2.65 mg/dL, likely in the setting of hypotension occurring the previous night and overnight. Initial labs were significant for a troponin I of 4.440 ng/mL (reference range: ≤0.034 ng/mL) and a lactic acid of 3.5 mmol/L (reference range: 0.5-1.9 mmol/L). Chest radiograph showed no acute pathology, and a CT renal was negative for obstruction. The patient was admitted, and the cardiology team was consulted at this time.

On further examination of the patient’s medical records, it was found that she had a drug-eluting stent placed in her right coronary artery five years ago and a subsequent left heart catheterization one year ago that was significant for a complete thrombotic occlusion of her left circumflex artery with an inability to traverse the occlusion during the procedure. As well, once the cardiology team was consulted and performed an extensive telemetry review, they agreed that the patient had sustained monomorphic VT and was cardioverted to normal sinus rhythm during her course in the emergency room, without any episodes of atrial fibrillation noted. The patient admitted to having identical symptoms of dizziness and palpitations two weeks prior to this event, the day before her hip replacement surgery. At this point, they continued the amiodarone infusion with the plan to adjust her oral amiodarone to 400 mg twice per day and then wean her to 200 mg daily in a week's time, with the caveat of holding all other QT segment prolonging agents. The cardiology team also added 12.5 mg of metoprolol tartrate. It was determined that her elevated troponin I was nondiagnostic in the setting of direct current cardioversion and that her EKG did not meet criteria for non-ST-segment elevation myocardial infarction. An echocardiogram was obtained, which was significant for an ejection fraction of 36% with global left ventricular hypokinesis, a dilated left atrium, mild to moderate mitral valve regurgitation, mild to moderate tricuspid valve regurgitation, and moderate pulmonary hypertension. It was determined that the run of VT was likely secondary to her chronic ischemic complete thrombotic occlusion in her left circumflex coronary artery. Due to an inability to intervene on the lesion during her last heart catheterization and the associated collateral vessels, it was decided that the patient would be treated with medical management for CAD.

The remainder of the patient’s hospitalization was uneventful, without any episodes of atrial fibrillation noted on her telemetry. She was discharged with the recommendation to follow up with cardiology in the outpatient setting to assess her need for an implantable cardioverter-defibrillator (ICD).

## Discussion

Accurately distinguishing VT from SVT with aberrancy is critical because a misdiagnosis can expose patients to inappropriate and potentially harmful therapies. In older adults with structural heart disease, VT accounts for the majority of wide-complex tachycardias, with prevalence reported above 80% [1]. Two of the most frequently used EKG-based tools are the Brugada and Vereckei criteria. The Brugada algorithm has high sensitivity and specificity but requires detailed QRS analysis that can be difficult to apply in urgent settings [2]. In contrast, the Vereckei approach simplifies interpretation by focusing on lead aVR, which makes it easier to use when rapid decisions are required [3]. Both methods, however, become less reliable in patients with conduction disease, prior infarcts, or pacemakers, all of which are common in the elderly [4].

The Brugada and Vereckei algorithms remain the most studied strategies for evaluating wide-complex rhythms. The Brugada system uses a four-step process that begins with assessment of RS complexes in the precordial leads, followed by the R-to-S interval, evaluation for atrioventricular (AV) dissociation, and analysis of QRS morphology [2]. If no RS complex is present in any precordial lead, VT is diagnosed. If an RS complex is present and the R-to-S interval is greater than 100 ms, this supports VT. The presence of AV dissociation further favors VT, while the final step evaluates QRS morphology in leads V1 and V6 to identify characteristic ventricular patterns. Sensitivity has been reported as high as 98% and specificity around 96%, but the algorithm’s complexity can limit its practicality in urgent clinical situations [1,5]. Vereckei et al. introduced a simpler alternative that relied solely on lead aVR, examining the initial deflection of the QRS and the sequence of ventricular activation [3]. This method is quicker to apply and particularly helpful in emergency settings where time is limited. In practice, neither system should be treated as definitive, and clinical context must always guide interpretation.

The reason to use the Brugada and Vereckei criteria is not just to detect VT, but also to avoid giving inappropriate medications in the setting of atrial fibrillation with preexcitation, such as in Wolff-Parkinson-White syndrome. In these cases, the wide-complex tachycardia is due to conduction through an accessory pathway rather than VT. If misdiagnosed, administration of AV nodal blocking agents, such as beta-blockers, calcium channel blockers, digoxin, or adenosine, can paradoxically accelerate ventricular response by allowing preferential conduction down the accessory pathway, potentially precipitating ventricular fibrillation. For this reason, antiarrhythmics that act on both atrial tissue and the accessory pathway are preferred. Intravenous procainamide is a recommended first-line therapy, while class Ic agents such as flecainide or propafenone may also be used in select patients [6].

Chronic CAD often leads to structural changes in the heart that promote VT. Repeated ischemic injury leads to fibrosis and scar formation, which in turn creates slow conduction pathways that allow reentry circuits to develop [7]. These border zones, often associated with prior infarcts or chronic occlusions, become the source of sustained monomorphic VT [8]. In our patient, the complete thrombotic occlusion of the left circumflex artery represents the type of unrevascularized scar that promotes reentry circuits and predisposes to monomorphic VT. Even when collateral vessels provide flow, conduction remains heterogeneous, and arrhythmogenic potential persists for years. A reduced left ventricular ejection fraction (LVEF) is a strong marker of risk, since it reflects the degree of scarring and remodeling [9]. Chamber dilation and valvular dysfunction add further instability [10]. Chronic ischemic cardiomyopathy, therefore, carries a high risk of recurrent VT and sudden cardiac death, making recognition of prior ischemic injury crucial for long-term planning and device decisions [11].

Amiodarone is central in the management of VT, particularly for patients with structural heart disease in whom other agents may trigger dangerous arrhythmias [9]. Intravenous amiodarone is highly effective for acute stabilization, with reliable termination of VT in both ischemic and nonischemic cardiomyopathy [12]. Oral therapy helps maintain rhythm control and is safer than class Ic drugs, which are contraindicated in ischemic disease, or sotalol, which increases the risk of torsades de pointes [13]. QT prolongation remains an important limitation and requires continuous EKG monitoring and strict avoidance of other QT-prolonging drugs [14]. In this case, the patient developed QT prolongation following cardioversion, emphasizing the importance of careful monitoring during initiation. Long-term use of amiodarone is also associated with thyroid disease and pulmonary fibrosis, so patients require ongoing surveillance [15]. This balance between efficacy and toxicity highlights the complexity of its role in both acute and chronic management.

Troponin elevation after cardioversion or prolonged tachyarrhythmia often reflects myocardial injury rather than infarction. True infarction requires both a rise or fall in troponin and evidence of ischemia through symptoms, EKG changes, or imaging [16]. Echocardiography helps identify ventricular dysfunction and wall motion abnormalities, while telemetry tracks rhythm stability, recurrence, and treatment response [17]. Although algorithms such as Brugada and Vereckei assist with rhythm interpretation, their reliability decreases in the setting of conduction disease or scarring [2,3]. Misinterpretation can occur with rate-related aberrancy, baseline bundle branch block, or old infarct changes [1]. In cases of uncertainty, especially among older patients with structural disease, guidelines recommend treating the rhythm as VT until further evidence becomes available through labs, EKGs, or imaging [16,17].

Patients with structural disease and reduced systolic function require long-term planning. ICD implantation is recommended for those with an LVEF of 35% or less after medical optimization, provided life expectancy is greater than one year [17]. Our patient’s LVEF of 36% places them just above this threshold, which highlights the importance of reassessment after optimization of medical therapy. Trials such as MADIT-II and SCD-HeFT demonstrated a survival benefit with ICDs in ischemic cardiomyopathy [18,19]. However, in older patients, frailty, comorbidities, and competing causes of death reduce the benefit, and decisions must be individualized. The DANISH trial further suggested that age and nonarrhythmic mortality limit the protective effect of ICDs even when arrhythmic risk remains high [20]. Guideline-directed medical therapy remains essential, including beta-blockers, angiotensin-converting enzyme inhibitors or angiotensin receptor blocking agents, mineralocorticoid receptor antagonists, and sodium-glucose cotransporter 2 inhibitors, which improve survival and reduce arrhythmias [21]. Statins and antiplatelet agents add further ischemic protection [22]. In borderline cases, wearable defibrillators, catheter ablation, and close reassessment can help refine long-term management [17,21].

Ultimately, algorithms such as Brugada and Vereckei are most helpful when paired with a clinical context that includes age, history of ischemia, conduction abnormalities, and hemodynamic status [1-3]. Prior infarction or chronic occlusion continues to promote arrhythmias long after the event through scar-related reentry [7,8]. Reduced LVEF remains a critical marker for risk stratification and device planning [17]. In elderly patients, polypharmacy, conduction changes, and electrolyte disturbances further increase the risk of torsades when using QT-prolonging medications like amiodarone. Careful medication review, EKG monitoring during initiation, and frequent reassessment of the risk-benefit balance are all required [14,23]. When diagnostic uncertainty remains, the safest approach is to treat the arrhythmia as VT until additional information becomes available [1,17].

## Conclusions

This case emphasizes the importance of prompt rhythm identification in elderly patients presenting with wide-complex tachycardia. Application of validated algorithms such as the Brugada and Vereckei criteria allowed for accurate recognition of VT, leading to timely cardioversion and stabilization. The patient’s chronic ischemic occlusion underscores the strong relationship between unrevascularized coronary disease and the development of scar-related reentrant circuits. Amiodarone provided effective rhythm control but required careful monitoring due to its association with QT prolongation. Ultimately, this case highlights the need for early differentiation of tachyarrhythmias, recognition of chronic ischemic substrates, and thoughtful selection of antiarrhythmic therapy as cornerstones of safe and effective management.
